# Mindfulness Practices in borderline personality disorder: A review of the literature

**DOI:** 10.1192/j.eurpsy.2023.2058

**Published:** 2023-07-19

**Authors:** C. Mariem, M. Gros

**Affiliations:** 1 Le Mans France, sarthe public mental health establishment, Le Mans, France

## Abstract

**Introduction:**

*Borderline personality disorder* (*BPD*), also known as emotionally unstable personality disorder is a severe disorder of emotional regulation. In people with BPD, mood swings are extreme, relationships are uncertain, and emotions are difficult to control, suicide and self-destructive behaviors are extremely common. medical treatment can certainly reduce the symptoms and suffering of people with BPD, but it is still not enough. The treatment is mainly based on psychotherapy especially **Dialectical behavior therapy (DBT)** focuses on the concept of **mindfulness**, or paying attention to the present emotion.

**Objectives:**

To assess the current level of evidence for mindfulness in BPD.

**Methods:**

a systematic review was performed using the database PubMed / Medline, using the following keywords: “MCBT”; “DBT”; “Mindfulness Therapy”;” BPD”; “Borderline personality”.

**Results:**

Research shows that the mindfulness therapy approach teaches skills for controlling intense emotions and reducing self-destructive behaviors. Decentering appears to play a crucial role in the treatment as a primary mechanism of action in this therapy.

**Conclusions:**

results suggest that the Mindfulness therapy is a main component for BPD treatment.

**Disclosure of Interest:**

None Declared

